# Recent and emerging food packaging alternatives: Chemical safety risks, current regulations, and analytical challenges

**DOI:** 10.1111/1541-4337.70059

**Published:** 2024-11-27

**Authors:** Charlene Lacourt, Keya Mukherjee, Jossie Garthoff, Aaron O'Sullivan, Leo Meunier, Vittorio Fattori

**Affiliations:** ^1^ Danone, Food Safety Center Danone Nutricia Research B.V. Utrecht The Netherlands; ^2^ Food and Agriculture Organization of the United Nations Rome Italy

**Keywords:** active and intelligent packaging, chemical hazards, food packaging alternatives, recycled plastics, risk assessment

## Abstract

Food contact materials should not release their constituent substances into food at levels harmful to human health nor change the food composition, taste, or odor unacceptably. The historical evolution of food packaging shows that the use of plastics has increased dramatically, because of its convenience, lightweight, and cost effectiveness, but carries a significant environmental impact. Influenced by trends such as growing awareness of the environmental footprint and stricter safety requirements, conventional packaging is now progressively evolving toward new alternatives. All stakeholders in the agrifood system are involved in the journey to transform food packaging to more sustainable alternatives, while maintaining the important functionalities of suitable food packaging. The current most promising food packaging alternatives are presented in this review with their benefits, limitations, and associated potential safety hazards, with a focus on chemical hazards. Although some potential hazards are common to conventional packaging, others are specific to the new alternatives. Identification of potential chemical hazards associated with these new packaging alternatives is important to anticipate any risks posed to consumer safety. With much diversity in packaging types and rules aimed at ensuring safety drastically varying between jurisdictions, it is not always easy to determine the best way to assess the safety of food packaging. International guidance on principles for safe food packaging could help drive global harmonization and would play a crucial role in ensuring a consistent and science‐based framework for the safety and compliance of new and emerging food packaging.

## 1 INTRODUCTION

Food contact materials (FCM) and articles refer to the materials and objects that are meant to come into contact (directly or indirectly) with food, that is, through equipment, containers, packaging, and various utensils. They are intended for the manufacture, preparation, preservation, transport, and handling of food products (Tsochatzis, 2021). In principle, FCM must be sufficiently “inert” to protect the integrity of the food they contain while ensuring that substances do not transfer from FCM into food at levels which change the organoleptic properties of food or endanger human health in the process (Ariosti, 2017; de la Cruz García et al., 2023). This review focuses on FCM used as food packaging, which has an essential role within the food chain.

Most foods and especially, manufactured, or prepared foods are sold packaged in some fashion. The primary functions of food packaging are to contain food products, ensure appropriate hygiene, promote easy handling, and transport and protect food from outside influences such as contamination (microorganisms, environmental hazards, etc.) between production and consumption stages (Han et al., 2018). By presenting a place for labels, packaging facilitates consumer interactions providing information on the product such as the identity of the food product, the composition (e.g., nutrition, food allergens), safe preparation, and storage instructions. Packaging can also be used for food traceability purposes. These features of food packaging are fundamental aspects for protecting consumer safety (Marsh & Bugusu, 2007).

While food packaging has been used for many centuries to protect and deliver food to consumers, it is only around the early 1960s that laws emerged in several jurisdictions to control the fundamental FCM requirements for protecting the integrity of food and human health (European Commission, 1976). From the 1980s to 2000, food packaging manufacturers developed a much greater understanding of the chemical composition of FCM and the nature of the substances that may be able to transfer from FCM into the food contained—a phenomenon called migration. This process can occur during production, handling, storage, and distribution of food and therefore has consequences for food safety and consumer health (de la Cruz García et al., 2023). Since then, the chemical composition and use of specific substances in FCM have been subject to tighter regulatory controls in countries and regions.

A great diversity of materials—mainly plastics, paper and cardboard, glass, and metals—can be used to manufacture food packaging depending on the desired physical and technical features from rigid (bottles, trays, cans, jars, caps), semi‐flexible (caps, closure, boxes) to flexible (bags, wraps, squeezable tubes). The choice of the packaging material is also guided by its intrinsic properties and suitability for the desired food or beverage. To enhance the functionality of the final packaging, sometimes several materials are combined to integrate their inherent properties to develop multi‐material packaging or laminates. In terms of global usage, some estimates report that paper and cardboard represent 34% of materials used for food packaging, with glass and metals corresponding to 11% and 6%, respectively (Severin et al., 2023). However, it is the introduction and rise in plastic usage in the last century that has transformed the packaging landscape dramatically, with plastics reaching 37% of materials used for food packaging. This growing demand for plastics is driven by the different characteristics they provide—lightweight, resilient, cheap, and modular (Severin et al., 2023).

Despite the immensely important role of food packaging in keeping food safe and reducing food waste, food packaging can also be associated with a negative environmental footprint. This is due to the petrochemical origin of certain packaging materials, their high production volumes, short usage time, and subsequent end‐of‐life waste generation coupled with inadequate management measures put in place to capture and recycle food packaging materials (Ncube et al., 2020). The use and contemporary awareness of some controversial substances, such as per‐ and polyfluoroalkyl substances (PFAS), phthalates, bisphenols, and mineral oils in food packaging have resulted in consumers becoming wary of FCM and the associated potential negative impacts on their health and the environment (BEUC, 2023; Lorenzini et al., 2010; Ramírez Carnero et al., 2021). Therefore, there are efforts, both at the level of relevant authorities and food and beverage industries, to bring forth safer and sustainable alternatives to some of the conventional packaging materials.

As the search for alternative packaging progresses, especially due to the attention to circular economy, it is vital to carefully consider the potential food safety issues associated with the alternatives. This review provides an overview of the main trends that have driven the development of promising food packaging alternatives. For each emerging alternative, a description is provided followed by a discussion of the potential chemical safety risks for human health associated with them and the corresponding current regulatory frameworks. The challenges associated with analytical methods to identify and detect the potential safety hazards in FCM are also addressed.

## 2 TRENDS INFLUENCING FOOD PACKAGING AND FUTURE DEVELOPMENTS

To qualify as a suitable and functional food packaging, the material needs to fulfill four general roles and functionalities (Ariosti, 2017; Marsh & Bugusu, 2007):

1. Provide a proper barrier function against external chemical, biological, and physical hazards to protect and preserve the quality and safety of the food it contains.
2. Facilitate food storage, distribution, and transport, thus also supporting the reduction of food waste at various stages of the supply chain.
3. Provide information to consumers on the composition of the contained food product, including its nutritional value, best before date, use instructions (e.g., serving size), food manufacturer details, and other legal requirements (e.g., allergen declarations) as applicable in the country of sale.
4. Be safe and suitable for its use as packaging in contact with food without releasing packaging‐derived substances that can pose a risk to human health, nor modify the nutritional composition, or cause undesirable changes in the sensory characteristics of food.

The use of food packaging materials is expected to continue to increase over the next decades. Despite the growing awareness among the public and policy makers of the environmental and health impacts linked to the production of conventional food packaging, packaging usage and the resulting waste at the end of life is still rising. The increasing attention and demand of consumers regarding the importance of implementing circular economy however plays a key role in shaping the food packaging landscape with various initiatives, introduced at global, regional, and national levels, to tackle some of the major issues with food packaging. Such trends are galvanizing efforts to explore different alternatives to conventional food packaging.

### 2.1 Growing awareness of the environmental footprint of food packaging

For all types of packaging, issues around large production volumes, waste generation, insufficient recycling, contribution to greenhouse gas emissions, material persistence, and/or degradation in the environment have led to growing concerns about the adverse environmental impacts. Reduction of the environmental footprint of food packaging has gained a lot of attention in the last decade from policy makers to food manufacturers and consumers.

Each year, around 150 million tonnes of plastic are used globally to produce various types of packaging materials with 37 million tonnes dedicated to food packaging for the agricultural value chain alone (FAO, 2021a; Novakovic et al., 2023; PlasticsEurope, 2022). If current trends continue, the production of primary plastics is expected to increase considerably in the coming years (OECD, 2022b). Plastic production itself has significant impacts on the environment, such as contributing to 3.4% of global greenhouse gas emissions throughout their lifecycle (OECD, 2023). Half of all plastic produced is designed for single‐use purposes. Millions of tonnes of plastic waste are lost to the environment, or sometimes shipped thousands of kilometers to destinations where it is mostly burned or dumped (UNEP, 2023b). According to estimates, 264 million tonnes of paper and cardboard were used to produce all‐types packaging in 2021 (Statista, 2023). No data were found on the amount of paper and cardboard dedicated to food packaging. The use of paper and cardboard‐based materials has an appreciable environmental impact as it contributes to deforestation (when virgin wood is used), requires energy to be produced, and contributes to pollution through waste generation (Stravens, 2023). However, as paper and cardboard have the advantage of being bio‐based, biodegradable, and recyclable, their environmental footprint is considered lower than other materials such as plastic (Oloyede & Lignou, 2021). Metals and glass also represent a significant volume of food packaging. The environmental impacts associated with the production and recycling processes of glass and metals are not fully determined (GPI, 2023; MPE, 2023). Based on the growing awareness around pollution from packaging waste, there are initiatives to substitute certain materials with other packaging alternatives, which are presented in the next section. However, environmental considerations around such substitutions must be carefully weighed. For instance, while glass‐based packaging has a lower environmental footprint than plastic, the former can be significantly heavier than the plastic counterparts and therefore can have higher transportation emissions (Abejón et al., 2020).

One of the main environmental burdens associated with the waste of certain food packaging is their persistence in the surroundings as they break down into progressively smaller pieces that can accumulate in the ecosystem and in animals (Ncube et al., 2020). While microparticles from metals and glass exist in our environment and must be addressed accordingly, it is the issue of micro‐ and nanoplastics that poses a greater concern simply due to the widespread use of plastics in articles (cosmetics, textiles, equipment, automobiles, fishing nets, among others) used in everyday life that go beyond food packaging. Most plastics degrade extremely slowly and break down into progressively smaller pieces. There is a lack of consensus with regards to the definition and size ranges associated with macroplastics (>5 mm to 100 cm), microplastics (>0.1 µm to <5 mm), and nanoplastics (1–100 nm) (Chamas et al., 2020; EFSA, 2016a; FAO, 2022a; Hartmann et al., 2019; Kumari et al., 2022; WHO, 2022).

To tackle the environmental issues associated with food packaging, efforts are being made to apply circular principles to food packaging. While the 3Rs (reduce, reuse, and recycle) approach is generally associated with circular economy, food packaging solutions need to focus on adopting a reduce–reuse–recycle–redesign (4R) approach, particularly aiming at minimizing the need for single‐use plastics, encouraging the reuse and recycling of materials while at the same time improving the production quality of food packaging from the design stage (FAO, 2021b; Pearson et al., 2024).

An important part of the packaging reduction strategy is avoiding and rationalizing unnecessary packaging which depends on the food product and the way it is produced, processed, and transported (Ma et al., 2020). Some secondary packaging can be redundant and certain food commodities (e.g., grains, nuts, beans, certain fruits, and so on) could be sold without packaging (FAO, 2021b). Moreover, packing larger portions instead of individual smaller portions can help to reduce the amount of packaging. The trend of local production and consumption can shorten distances that a food will have to travel from farm to fork, and this may lead to opportunities to effectively reduce the amount of packaging usually needed for long‐distance transport and storage. These are reviewed in detail elsewhere (Dörnyei Krisztina et al., 2023; Ma et al., 2020; Pearson et al., 2024; Versino et al., 2023; Wilson et al., 2017; Yokokawa et al., 2021).

Amid the growing awareness about the environmental impact of food packaging, particularly plastics, many initiatives have been taken at global and national levels. These initiatives aim to reduce packaging waste while encouraging the use of safe and more sustainable options. A global overview of some of these initiatives has been summarized in Table 1. Even if the level of commitments and timelines differ between regions and countries, the shared goals are to drastically reduce the use of single‐use plastics and to reach defined targets for the percentage of recyclable, recycled, or reusable plastics. Besides the initiatives taken at global and national levels, more than 500 companies (representing 20% of all plastic packaging produced globally) have committed to the use of 100% recycled, recyclable, or reusable plastic packaging in the coming years through the New Plastics Economy Global Commitment launched by the United Nations Environment Programme and the Ellen MacArthur Foundation. The commitment brings together businesses, governments, and other stakeholders to begin working toward building a circular economy for plastics (Ellen MacArthur Foundation & UNEP, 2023).

**TABLE 1:** Some key initiatives across the world to tackle environmental impact.

Organization/region/country	Commitments and initiatives	References
United Nations Environment Program	Historic resolution by member countries in 2022 to forge an international legally binding agreement to address global plastic pollution.	UNEP (2022b)
European Union	Adoption of the Single‐Use Plastics Directive in 2019, with new rules adopted in 2022 (such as polystyrene banned as a single use plastic). A chemicals strategy for sustainability on October 14, 2020. This is part of the EU's zero pollution ambition, which is a key commitment of the European Green Deal. A new agenda for sustainable growth in the new Circular Economy Action Plan (CEAP) adopted in 2020. The EU's Plastics Strategy, part of the EU's CEAP, contributes to reaching the 2030 Sustainable Development Goals by aiming at reducing single‐use plastics and encouraging reuse and recycling. In 2023, the European Parliament adopted its position on the Packaging and Packaging Waste Regulation (PPWR) (measures to reduce packaging with set targets, limit specific types and ban of some chemicals such as PFAS and BPA in food packaging) and several initiatives on microplastics. The Ecodesign for Sustainable Products Regulation (ESPR), which entered into force on 18 July 2024. A key part of EU's approach to ensure more environmentally sustainable and circular products.	European Commission (2019) European Commission (2020a, 2020b) European Commission (2020a, 2020b) European Parliament (2023) European Parliament (2024)
United States of America	The National Recycling Strategy reaffirms the goal to increase the recycling rates of various materials, including plastics, to 50% by 2030 The draft National Strategy to Prevent Plastic Pollution focuses on actions at eliminating the release of plastic waste from land‐based sources into the environment.	EPA (2023a) EPA (2023b) US Department of State (2022)
China	The 14th Five‐Year Plan (2021–2025) includes eliminating single‐use plastics, encouraging recycling and promoting alternatives to plastics, and developing and adopting more circular product designs and systems.	Yee (2023)
Japan	The Plastic Resource Circulation Act came into effect in 2022 that aims to improve plastic resource circulation by all entities involved in accordance with circular economy principles.	Government of Japan (2022)
South America	A Global Environment Facility (GEF) funded project “Reduce marine plastics and plastic pollution in Latin American and Caribbean cities through a circular economy approach” was launched in 2022. Initiatives at country level include the Colombia Plastics Pact in Latin America launched in 2023 and a draft outlining a ban of single‐use plastics products made from noncompostable materials in Brazil.	GEF (2022) ICIS (2023)
Australia, New Zealand, and the Pacific Island countries	The Australian, New Zealand, and Pacific Islands Plastics Pact (ANZPAC), launched in 2021, aims to reach certain objectives by 2025: the phase‐out of problematic and unnecessary single‐use plastic packaging; to make 100% of packaging reusable, recyclable or compostable; to increase the effectiveness of recycling by 25% (at least).	ANZPAC plastics pact (2021)

### 2.2 Greater attention to the safety of the chemical constituents of food packaging

Next to the environmental considerations, there are also potential food safety risks associated with the presence of certain substances (or their breakdown products) in the FCM that can migrate from the packaging material into food. This migration of chemicals may alter the quality and flavor of food as well as impact food safety (Hahladakis et al., 2018). Migration depends on several properties of the migratory substances such as molecular weight, affinity to the material and/or to the food, and the concentration of the substance in the material. Other relevant factors influencing migration are the presence of functional barriers, temperature and pH of the food contained, time of contact between packaging and food, and conditions of storage (Arvanitoyannis & Bosnea, 2004). If an FCM contains substances that can migrate into the food contained, the safety of the FCM needs to be demonstrated for the intended use. Recent advances in research and analytical methods have allowed for the generation of more toxicological data and the identification of more migrating substances, thereby increasing our knowledge of potential safety risks associated with food packaging (ILSI Europe, 2023).

In general, intentionally added substances (IAS) and non‐intentionally added substances (NIAS) are the two main classes of substances associated with FCM. The IAS include those that are specifically added to packaging in the manufacturing process for a specific purpose, such as monomers used for construction, additives (such as antioxidants, UV‐absorbers, and photo‐initiators) to improve characteristics, and so on. The NIAS can be impurities from IAS or products created during the manufacturing, reaction products, or degradation of the material. Migration of various substances, both IAS and NIAS, have been documented in literature, such as antioxidants and colorants from laminated packaging, or oligomers from polyethylene terephthalate (PET) and can coatings, among others (Forooghi et al., 2022; Terrasse et al., 2022; Ubeda et al., 2018). On the other hand, the migration of compounds from packaging can also be leveraged to ensure the maintenance of the safety and quality of food products contained (EFSA, 2013). For instance, substances with oxygen‐scavenging functions are intentionally released from the packaging into food to extend the shelf‐life of the food while being safe for the consumer, as the amount of the substance released is controlled and evaluated for consumer safety. It should be noted that the level of substances migrating from food packaging is often low to very low and is usually far below a safe migration level. But it may potentially become a health concern when the sum of all migrating substances with similar modes of action is considered. Multiple IAS and NIAS can in theory migrate together—though at minute amounts—and be consumed. Although there are no official risk assessments that specifically tackle food safety risks from exposure to multiple chemicals migrating from FCM, there are some emerging guidance documents and positions (EFSA, 2021a; FAO & WHO, 2019).

Certain migrating substances, commonly used for their grease‐proofing functionalities, for example, members of the PFAS family, certain phthalates, and mineral oils, among others are considered to represent critical health hazards and could be associated with health concerns (Geueke & Muncke, 2018; Lorenzini et al., 2010; Ramírez Carnero et al., 2021). Some of the possible human health impacts include genotoxic potential, toxic effects on the endocrine, reproductive, and developmental systems and impact on immunity (EFSA, 2023; Wang & Qian, 2021). Certain populations like infants and young children are particularly vulnerable to the effects of these substances, due to their immature organ system and highest exposure on a body weight basis (Hulin et al., 2014). While adverse health impacts linked to bisphenol A (BPA) and its safe exposure limit are still being discussed, several countries and regions (United States of America, Canada, Australia, New Zealand, China, and the European Union) have already moved to restrict its use, particularly for infants and young children.

### 2.3 Evolution of FCM regulations to protect both human health and the environment

From the 2000s, the regulatory landscape has seen the defining and refining of standards for FCM, with the chemical composition of FCM controlled in a fashion similar to food additives, resulting in positive lists of food contact substances that have undergone a battery of toxicological tests based on their anticipated use and migration levels. Today, regulations on FCM around the world remain quite heterogeneous. This ranges from countries without any specific regulation on FCM to countries where national and supranational regulations apply (de la Cruz García et al., 2023). In regions or countries that do have regulations for FCM, two overarching regulatory approaches exist. One focuses on controls on the finished product (i.e., the final food contact article and the food packed inside) using migration testing with food simulants to check for compliance. This model is followed by most of the regions and countries (e.g., Europe, Latin America, and China) that have regulations for FCM. In these regions, the compliance responsibility is shared all along the food chain, with the supplier responsible for delivering a compliant product and the customer responsible for verifying it. The other regulatory approach focuses on the quality control of the material used in the packaging manufacturing with compliance responsibility lying with the article producers. In countries that use this approach (e.g., United States of America and Canada), each new packaging application is directly validated by relevant authorities.

FCM regulations overseeing the management of consumer safety have existed for decades. However, in recent years, there has been an increasing number of regulations aimed at reducing the environmental burden linked to food packaging and packaging waste in addition to improving food safety and public health. Certain jurisdictions, such as described in Table 1, have developed various general and targeted policy interventions to tackle this growing environmental issue. The major principles behind these policy efforts include setting national targets, prioritizing interventions according to needs, and enabling a circular economy or life cycle approach to packaging (UNEP, 2023a). Also, with rising awareness about the risks of chemicals leaching from FCM degradation into the environment, several initiatives have been taken at various national or supranational levels to restrict the application of certain types of FCM that can persist in the environment such as PFAS‐based materials or polystyrene. While new regulations aimed at reducing the environmental impacts of food packaging are essential, developing products that comply with regulations both in terms of ensuring consumer safety and reducing environmental impacts can bring additional challenges for the food industry. For example, the composition of bio‐based or recycled materials can vary a lot depending on the diversity of feedstock and therefore be harder to control than the composition of commonly used virgin materials for which there is more information. Considering this, additional controls or steps in the process must be carried out to use emerging sustainable materials while ensuring consumer safety. The future European regulation for FCM to be published in the coming years is expected to provide a framework to ensure the safety of sustainable FCM.

### 2.4 Evolving public perceptions toward food packaging

Due to growing awareness of circular economy principles, consumers are concerned about food packaging waste and the related negative impacts on the environment, and are expecting the food industry to provide sustainable alternatives to plastic packaging (Brennan et al., 2023; Fooddive, 2020; Otto et al., 2021). The results of a study carried out in Canada show that responsible packaging (i.e., recyclable, reusable, or compostable) is perceived to have a positive correlation with the natural and healthy character of the product contained (d'Astous & Labrecque, 2021). Moreover, consumers expect food to be of better quality and safety when eco‐labels are present on the packaging, irrespective, of the contents of the label referring to the food or the packaging. Glass, paper, and cardboard packaging are perceived to be the healthier and eco‐friendly packaging options, while plastic trays are considered as the least appropriate (Food Packaging Forum, 2020). Consumers show interest in purchasing innovative packaging, preferring sustainable packaging over others like intelligent packaging (Cammarelle et al., 2021). However, public knowledge about the practical implementation of recyclability, biodegradability, and reusability of packaging remains low (Otto et al., 2021).

While consumers tend to rely on regulatory bodies and the industry to ensure food packaging safety, the awareness among consumers about the health impacts of packaging, particularly the presence of certain chemicals such as phthalates, BPA, and PFAS, is increasing (VZBV, 2020). In a European survey of 26,500 respondents, half the participants had heard of substances that are able to migrate from food packaging (EFSA, 2022). Even if their knowledge about potential risks linked to food packaging is low, most consumers value the safety and quality of food packaging as important criteria when they buy food (Bou‐Mitri et al., 2021; Macena et al., 2022). Another European study of 11,200 people highlighted the consumer concerns around food packaging, particularly the fact that they do not feel sufficiently informed about the packaging when buying a food commodity, either in terms of the environment or health impacts such as directions for safe and appropriate use. About 90% of the study participants supported stricter rules on packaging to prevent adverse health impacts (BEUC, 2023).

### 2.5 Applications of food packaging expected to grow

Food loss (i.e., occurring along the food chain except at the retail level) and food waste (i.e., occurring at the retail and consumption levels) carry a huge environmental burden in the form of depleted resources (water, soil, energy, and so on) that go into producing and making food available to consumers (FAO, 2013). According to estimates, approximately 14% of the total food produced is lost along the way and 17% of total food is wasted at the consumer level contributing to food insecurity (FAO, 2019; UNEP, 2021a). The role of food packaging in tackling these enormous issues is paramount, especially given the challenges associated with lengthening supply chains and the changing climate.

Contemporary technological innovations have further improved upon the desired functionalities of food packaging and have brought two new types of packaging: active packaging and intelligent packaging whereby the packaging itself is modified to interact with and monitor the food contained within to extend shelf‐life. Such emerging technologies, while necessary, create a paradoxical effect wherein to protect food from being lost or wasted, there is an environmental price to pay in terms of packaging production and use. In this context, some life‐cycle studies do indicate that the food matrix contained within a package can be associated with much greater environmental impact than the packaging itself (Miller, 2020; Williams & Wikström, 2011). While such sentiments are not widely recognized, it has been proposed that the scrutiny around the environmental impacts of food packaging should be more subjective, for instance, by also taking into account any resulting reduction in food waste (Heller et al., 2019).

It is estimated that a 70% increase in food production, relative to 2009 levels, may be needed by 2050 to keep up with rising population and incomes (d'Astous & Labrecque, 2021; FAO, 2011). This increase in food supply and rising globalization are expected to favor the need for more packaging. In fact, over the last few decades, there has been an increase in the consumption of prepared and manufactured food sold in some form of packaging. In addition, rapid urbanization and the COVID‐19 pandemic have greatly influenced the trend of consumers purchasing food (groceries, meal kits, food take‐away, among others) through online portals requiring tailored solutions for storage and transportation.

## 3 PROMISING FUTURE PACKAGING SOLUTIONS—OPPORTUNITIES AND FOOD SAFETY CHALLENGES

Exploration of potentially sustainable and safer alternatives to conventional food packaging is an active area for the packaging industry. Much of the discussions around the development of these alternatives are either focused “upstream” or preconsumer, such as material redesign, plastic reduction, and substitution of currently used food packaging or “downstream” focused on postconsumer solutions, such as recycling and reuse (The Pew Charitable Trusts, 2020). Currently, packaging alternatives tend to focus on reusing (recycled materials, refillable containers) or discovering materials that do not result in persistent waste (biodegradable packaging). While some of these alternatives are still under development, there are some already on the market used for packaging a variety of different foods. Like it is for conventional packaging, the type of foods suitable for new packaging alternatives will involve considerations such as properties of food, duration of storage, and economic viability, among others. Some of the promising emerging food packaging, their benefits, challenges, regulatory framework, and potential safety concerns are explained below and outlined in Table 2.

**TABLE 2:** Benefits, limitations, and potential safety hazards of some emerging food packaging alternatives.

Type of packaging	Currently on the market	Benefits	Limits	Potential safety hazards
Recycled packaging, e.g., metals, glass, plastics (rPET)	Glass Metals Plastics (PET in the European Union and the United States of America, Polyolefin in the United States of America) Paper (not in direct contact in most cases)	Contribution to circularity: RECYCLE Important impact on reducing plastic pollution Good properties to transport, store, and preserve food Good consumer perception	For all: energy consumption for recycling process For glass and metal: no specific limit For paper: safety not necessarily approved for direct food contact (only for packaging not in direct contact) For plastic: lack of sorting and recycling facilities beyond PET bottles, available FCM feedstock, limited number of recycling cycles, cost, use currently limited to certain types of plastics due to decontamination efficiency, not possible with multilayer packaging (when inseparable)	Potential sources of migrating substances: non‐food contact materials, anterior (mis)use, contaminants from substances used in the recycling process (e.g., detergent), degradation products from the material because of the recycling process (e.g., due to high temperature) Production of micro‐ and nano plastics
Reusable packaging, e.g., glass bottle deposit	Depositing glass bottles Metal bottle Glass/plastic cup for home‐made yogurts Paper sachets for (dried) fruits, cereals, and leguminous plants	Contribution to circularity: REUSE, REDUCE Important impact on reducing plastic pollution Good properties to transport, store, and preserve food Good consumer perception	For certain types of food: shorter timeframe for consumption to prevent microbiological contamination Consumer education around hygienic practices for using reuseable food contact articles	Microbiological contamination Potential sources of migrating substances: anterior (mis)use, washing step Higher migration levels due to longer duration of contact
Bio‐based packaging, e.g., PLA, Bio‐PET, edible materials	Paper/paperboard: broad use in the worldwide market for food packaging Bioplastics have limited applications, accounting for 1‐2% of all plastics used. Starches, PLA, and PBAT are more commonly used Edible packaging: no data available	Contribution to circularity: REDUCE Use of renewable sources Large range of possibilities with a large diversity of materials and properties similar to conventional plastic Good consumer perception	Impact of large‐scale production on environment and competition with the food and agriculture sectors for resources Energy consumption for production Lack of available facilities for recycling Potential shorter shelf‐life Higher cost than conventional packaging cost	Potential migrating substances specific to bio‐based materials: pesticides, allergens, natural toxins, persistent organic pollutants, phytoestrogens Synthetic additives Current analytical methods not adapted to detect substances specific to bio‐based materials (e.g., allergen above 1000 Da) Microbiological contamination of edible packaging
Active and intelligent materials and nanotechnologies, e.g., silver nanoparticles incorporated into plastic for fresh meat or seafood packaging	In the United States of America: active packaging account for 1% of total food packaging Major users of these technologies: Japan followed by the United States of America and the European Union	Contribution to circularity: REDUCE (by reducing food waste), REDESIGN Extend the shelf life of food Applicable to several types of material	Energy consumption for production More complicated waste management systems needed Higher costs involved in production than conventional packaging cost	Limited knowledge regarding toxicity of nanoparticles after oral exposure Intentional release of substances from packaging into food Hazard specific to the type of materials (e.g., specific to plastics)
Hybrid packaging, e.g., recyclable plastic container covered by printed removable cardboard	13% of all paper‐based packaging in the European Union Present in many countries but no other data found	Contribution to circularity: REDUCE, REDESIGN Reduce the fossil‐based parts while keeping the expected properties of the packaging	Energy consumption for production Could result in more difficult sorting for waste management. Layers need to be correctly separated by the consumer to enable an efficient sorting and recycling Could slow down the transition to fully sustainable alternatives	Migrating substances from external layers Hazard specific to the type of materials (e.g., specific to cardboard)

Abbreviations: FCM, food contact materials; PBAT, polybutylene adipate terephthalate; PET, polyethylene terephthalate; PLA, polylactic acid.

### 3.1 Recycled materials

One of the key public demands, and an unavoidable future for food packaging, is the expansion of the applications of recycled materials to revalorize resources already in circulation and in response to regulations in certain jurisdictions that limit landfilling of plastic waste.

#### 3.1.1 Description

Recycled materials, or more specifically postconsumer recycled materials include those that have served their intended uses having completed their life cycle as consumer items and have subsequently been discarded for disposal or recovery. After going through a process of washing and reshaping, the materials can be used again for new applications. Postconsumer recycled materials are an essential component to implement the circular economy for certain packaging types. However, the production of persistent food packaging waste can only be reduced, not prevented with recycling, due to a limited capacity for sorting and recyclability of different packaging materials (Brown et al., 2023).

Out of all the various packaging materials, glass has the longest history of recycling for food packaging followed by metals. When sorted correctly, both glass and metals can be qualified as permanent materials as they can be recycled almost indefinitely without significant changes to their composition and properties, except the accumulation of metal ions in certain cases that can be generally prevented by appropriate coatings (Geueke et al., 2018). The recycling of paper is quite high globally with more than 80% in European countries, 68% in the United States of America, and 46% in China. Virgin paper can be recycled up to seven times, with an average of 3.5 times. However, recycling may alter the quality of fibers and make them less suitable for materials in contact with foodstuff (e.g., potential increase in the levels of heavy metals) (Bandara & Indunil, 2022). Usually, recycled paper‐based materials are used as secondary food packaging or for other types of packaging.

Given the focus on the circular economy and several ongoing efforts across the globe to reduce plastic waste, the rate of collection and recycling of plastic remain insufficient to significantly reduce the impact of plastic waste pollution (UNEP, 2022b). High‐quality waste sorting for recycling is a challenge as packaging materials tend to be produced with a variety of material combinations and often get discarded into assorted trash post‐use. Plastics include diverse materials such as PET, polyethylene, polypropylene, polystyrene, polyvinyl chloride, polycarbonate, polyamide, and polyurethanes, but currently, only certain plastic materials, once collected and sorted, undergo recycling. Packaging materials can also be contaminated with food or other materials, making it difficult to clean and decontaminate for subsequent use (EFSA, 2011). The recyclability of a material is mainly based on the decontamination efficiency of the recycling process. Due to its low diffusion coefficient and its high melting temperature, PET (e.g., beverage bottles) remains the most recyclable plastic. The decontamination is more difficult for other polymers but not impossible. The number of authorized recycled polyolefin (e.g., polypropylene or high‐density polyethylene) applications for food packaging has increased in the United States of America within the last 5 years but they are not yet present in the European market. Generally, plastics that can be recycled can undergo the process only for a limited number of times. For such reasons, plastics tend to be downcycled into applications with less‐exacting specifications (i.e., from food grade to other consumer goods) than virgin materials (Tullo, 2019). Due to multiple challenges regarding collecting, sorting, and recycling, less than 10% of the seven billion tonnes of plastic waste generated globally has been recycled so far, while 19% was incinerated and almost 50% went into sanitary landfills. The remaining 22% were disposed of in uncontrolled dumpsites, burned in open pits, or leaked into the environment (OECD, 2022b; UNEP, 2022a).

In general, available capacities for recycling of plastics are heterogeneous with recycling channels not existing for each type of plastic in every geographical region (DeWeerdt, 2022; Waste 360, 2019). The types of recycling processes that exist today include—mechanical processes (i.e., extrusion and thermal decontamination or solvent extraction), chemical processes (i.e., pyrolysis or oxidation), and enzymatic processes. In terms of application, recycling by a mechanical process is the most predominant method for plastic recycling. Chemical and enzymatic recycling processes are designed to depolymerize materials into their respective monomers. This makes these processes attractive from a food safety perspective as they allow better control of the content and quality of the recycled materials. Shan et al. (2023) compared the environmental impacts of mechanical recycling and two types of chemical recycling (coke oven and gasification) and found that mechanical recycling showed lower total electricity requirements than chemical recycling methods. However, chemical recycling led to high‐quality products and byproducts that could be reused more effectively, reducing the overall environmental impacts of such technologies, and making mechanical recycling a less preferred option. However, the higher costs of the chemical and enzymatic processes have hampered their further development and large‐scale applications (Tullo, 2019).

#### 3.1.2 Potential food safety hazards

Considering the different measures outlined in Table 1, it is quite likely that most food packaging will include some recycled materials in the years to come. Given the current methods for producing recycled materials, the composition of recycled materials is less characterized compared to virgin materials. It is therefore important to control the quality of the feedstock and ensure efficient decontamination demonstrating that the level of contaminants that can migrate are kept as low as possible to protect consumer safety. When decontamination steps are not efficient enough, the contaminants potentially present in the recycled materials could come from different stages in the process:

‐ Anterior contaminants, arising from the previous use of the food packaging materials, can be carried over to recycled materials. These contaminants include residues from food contained previously in the packaging or results from the misuse of the packaging for non‐food applications. In the latter case, level of contaminants coming from previous misuse can be low, for instance, in the case of recycled PET intended for bottle‐to‐bottle recycling for direct food contact applications in Europe, contaminants arose from 0.03% to 0.04% of recollected PET bottles (3 bottles misused among 7000–10,000 bottles screened) (EFSA, 2011; Franz & Welle, 2022). Another source of contaminants could come from the small amount of non‐FCM allowed in the feedstock that also include nonauthorized additives. An example is the potential presence of brominated flame retardants, usually added to electronics and furniture, in recycled packaging (Paseiro‐Cerrato et al., 2021; Rani et al., 2014; Samsonek & Puype, 2013). Brominated flame retardants have been linked to adverse health impacts such as genotoxicity (ANSES, 2017; Turner et al., 2021). Contaminants can also arise in the feedstock from the materials usually present as layers behind functional barriers (not necessarily food contact compliant).
‐ Posterior contaminants, arising from the recycling process itself such as detergents used for washing the materials, new additives to obtain the desired properties of the final materials or contaminants arising from the degradation materials due to high temperatures used during the recycling process (EFSA, 2011; Gerassimidou et al., 2022; Möller et al., 2008). Improper sorting during the recycling process can introduce food safety risks through the presence of unwanted impurities. During the mechanical recycling of plastics, polymers are partially degraded by shredding and heated, which may result in the formation of unintended reaction products.

There is experimental evidence that recycled packaging contains chemical contaminants introduced during use, waste processing, and recycling and can migrate into packaged food. Unknown or hazardous substances may accumulate in recycled materials and could lead to human exposure if not carefully managed (Geueke et al., 2023). For instance, BPA that is not used for the manufacturing of PET was found as a contaminant in recycled PET (Dreolin et al., 2019). Scientific publications comparing chemical migration between virgin and recycled materials are however sparce. Those available tend to report an increased chemical migration from recycled materials compared to virgin materials, for instance, higher levels of antimony or benzene in recycled PET (Gerassimidou et al., 2022; Thoden van Velzen et al., 2020), higher amounts of degradation products of antioxidants in recycled polyolefins (Su et al., 2021), increased levels of oligomers in recycled PET (Geueke et al., 2018; López et al., 2014), and a greater number of diverse migrating substances in recycled paper (Lowe et al., 2021). Mineral oil hydrocarbons (MOHs) and heavy metals were also detected in recycled paper and recycled metals or glass, respectively (Focker et al., 2022). In a recent study, several genotoxicity bioassays conducted with migrating substances from recycled plastics (recycled polyolefins and polystyrene) highlighted that reactions with and degradation of printing inks formed during the recycling process could result in the presence of genotoxic substances (Mayrhofer et al., 2023). Regarding microbial risks, the high temperature used during the recycling process is considered sufficient to kill any microorganisms potentially present (Government of Canada, 2023; Marsh & Bugusu, 2007).

Methods to breakdown the plastics such as mechanical friction or abrasion within recycling processes may also increase the levels of microplastics and nanoplastics (MNP) in the wash water that is then subsequently discharged postrecycling (estimated microplastic count between 5.97 × 10^6^ and 1.12 × 10^8^ microplastics/m^3^ using fluorescence microscopy analysis). Adequate additional filtration steps may be needed to sufficiently control the volume of MNP discharged (Altieri et al., 2021; Brown et al., 2023). In terms of MNP release from recycled materials, while studies suggest that recycled PET is associated with the release of microplastic particles in water bottles, such observations were also reported for nonrecycled plastic bottles and carton beverages in the same study (Schymanski et al., 2018). While scientific evidence continues to grow, the varied impacts of microplastics on human health are still poorly understood with researchers and relevant organizations calling for greater standardization in exposure measurements (WHO, 2022). Recent studies demonstrate that, MNP are ubiquitous in the environment and that humans are exposed to a complex mixture (different sizes, shapes, polymer composition, etc.) through inhalation and a variety of food products, from seafood, drinking water (tap and bottled) to salt with other sources being routinely identified (Cverenkárová et al., 2021; Li et al., 2020; Pironti et al., 2021; Thiele et al., 2021). Once ingested (or inhaled), the translocation of MNP inside humans is largely dependent on their size, as smaller pieces (nano range) are more likely to cross‐intestinal barriers and end up in the bloodstream, increasing the potential risk for immunotoxicity and organ damage (FAO, 2022a; WHO, 2022). In addition, microplastics can also release plastic additives and unbound monomer residues into the environment. However, this is not considered to present a risk to humans through water or food if levels of exposure are sufficiently low (FAO, 2022a; WHO, 2022).

#### 3.1.3 Regulation

In Europe, the new EU regulation No. 2022/1616 on recycled plastics entered into force in 2022, replacing the former regulation 282/2008 and regulating all recycling processes with the purpose of ensuring safety of recycled plastics for food contact applications by requiring decontamination and high quality of the feedstock (>95% food grade). This regulation provides the legal framework for the development of any new recycled plastics obtained through a new recycling technology operational prior to a safety assessment by authorities (European Commission, 2022). Only recycled PET materials have been evaluated as suitable based on the former regulation. Within the new regulation, the recycling processes will be evaluated by the European Food Safety Authority (EFSA) after several months postmarket launch and once enough data are collected and positively assessed by EFSA, an authorization by the European Commission will be granted. There is no harmonized regulation for (recycled) paper and cardboard, metal, or glass in Europe. In the United States of America, recycled materials are evaluated by the Food and Drug Administration (FDA) and need authorization through a nonobjection letter before being placed on the market. In jurisdictions authorizing recycled materials for FCM like the European Union or the United States of America, the evaluation is based on the quality of feedstock, the decontamination efficiency of the recycling process, and the application of the recycled packaging (EFSA, 2008; US FDA, 2020). In China, South Korea, and Thailand, regulatory processes have either been initiated or are under development to evaluate the safety and subsequent authorization of recycled FCM.

### 3.2 Reusable packaging

#### 3.2.1 Description

The use of reusable containers is a promising solution in reducing waste associated with packaging. Reusable (or refillable) packaging refers to containers that, once emptied, can be washed, cleaned, and refilled with the same or different food products. This category can be divided into several types: refillable by a bulk dispenser, refillable parent packaging, returnable packaging, and transit packaging (Coelho et al., 2020). One of the classic examples is the deposition of glass bottles and follow‐up reuse systems that have existed for a long time across the world. With consumer demand and awareness of the challenges of conventional food packaging, there are currently various initiatives where manufacturers and retailers are offering more and more products that can be bought in bulk in the supermarket. For instance, installing refilling stations in supermarkets (marketed as “zero‐waste” stores) enables consumers to use their own washed/sterilized containers to fill them with food or non‐food items in bulk (e.g., cereals, yogurt, detergent, etc.). Depending on the reusability models used, there may be environmental impacts stemming from returning items to collection points, long‐distance transportation, the water footprint involved, source of electricity, and the number of times a specific item is used (UNEP, 2021b; Yadav et al., 2024). Another important point for consideration is that despite the name reusable containers cannot be used infinitely. Therefore, such packaging solutions can also generate waste which may need to be either recycled or disposed of appropriately.

#### 3.2.2 Potential food safety hazards

Consumer behavior strongly influences the food safety aspects of reusable packaging. Whether consumers are willing to adopt a different system and adhere to good practices when cleaning reusable containers at home will impact the safety of such packaging solutions. If consumers fail to adequately clean reusable containers, chances for cross‐contamination between uses increase. In such cases, there are microbiological risks to consider as such containers can not only contaminate the refilled food product but also potentially contaminate other people's food at the filling stations (Nahar et al., 2023). A particular food contact article encouraged for consumer use is reusable grocery bags. However, some studies have detected various foodborne pathogens (e.g., *Enterobactericeae*, *Listeria monocytogenes*) in such bags as they come into contact with meat and poultry or other fomites (Barbosa et al., 2019; Repp & Keene, 2012). Several steps have been suggested to prevent such food safety risks including washing the bags frequently, and to avoid using them for purposes other than food, and so on.

In addition, chemical contamination related to refillable packaging also needs to be taken into consideration. Polyamide and polypropylene are types of materials used for food packaging reported to be reused. Cyclic polyamide oligomers and degradation products of antioxidants such as 2,4‐di‐*tert*‐butylphenol are NIAS frequently found after extraction or migration from polyamide and polypropylene packaging, respectively (Geueke et al., 2023). These two NIAS may act as endocrine disruptors at certain levels (ECHA, 2023). Cleaning agents used to wash refillable packaging between subsequent uses could result in the adsorption of substances from cleaning products to the packaging or/and the deterioration of the packaging materials that can potentially lead to an increase of migrating substances into the food product. Certain chemicals present from previous use or even from consumer misuse may lead to potential food safety risks. According to Cavazza et al. (2021), polymeric chains in plastic materials can undergo progressive degradation after prolonged use in addition to mechanical, thermal, and chemical stresses, a combination that can increase the level of migration of certain substances. Other studies show an increase in the release of BPA and some NIAS (degradation products and traces of colorants) from aged and reusable containers and tableware made from polycarbonate as opposed to unused ones (Bignardi et al., 2015, 2017).

Furthermore, reusable packaging may result in longer contact between the materials and the food possibly leading to greater migration from packaging into food. Therefore, as a point of consideration, reusable packaging that is meant to be refilled with different types of food and beverages over a certain time must be evaluated for all potential substances that can migrate under various conditions of use (i.e., with food simulants covering most foods and repeated migration testing). However, this recommended testing method may not reflect the exact usage in real life, as it does not consider the washing step that can result in the adsorption of chemicals or degradation of the materials (Geueke et al., 2023; Tisler & Christensen, 2022).

#### 3.2.3 Regulation

There is no specific regulation for reusable packaging, but specific migration testing requirements for plastic packaging for repeated use apply in different geographical areas. In the European Union, migration testing must be performed three times for plastic packaging meant for repeated use with migration levels shown not to increase (European Commission, 2011). In the United States of America, specific procedures for migration testing must also be followed for food packaging meant for repeat uses (US FDA, 2007).

### 3.3 Bio‐based materials

#### 3.3.1 Description

Given the current emphasis on sustainability and circular economy, the area of bio‐based materials as an alternative to conventional plastics in packaging is gaining traction (Baranwal et al., 2022; J. Wang et al., 2022). In general, bio‐based materials can be categorized as bio‐based polymers and bio‐based fibers.

‐ Bio‐based polymers, either bio‐sourced or biodegradable or both, can be collectively referred to as bioplastics. These generally fall into four categories (European Bioplastics, 2018; J. Wang et al., 2022).
‐ Polymers extracted from biomass such as polysaccharides (starch and cellulose), proteins (chitin, collagen, casein, and soy proteins), and lipids.
‐ Synthetic polymers from natural substances. These include polymers such as polylactic acid where the monomers have a biological origin or polymers such as biopolyethylene where the monomers are produced chemically from bio‐sourced ethanol in this case. Such polymers are not necessarily biodegradable.
‐ Polymers produced by microorganisms (such as polyhydroxyalkanoates).
‐ Biodegradable polymers synthetized from petrochemical monomers (such as poly *ε*‐caprolactone), polybutylene adipate terephthalate).

Polymers from biomass provide some of the functionalities of plastics while being manufactured from renewable sources (Baranwal et al., 2022; Fera Science Ltd, 2019). However, bioplastics often lack certain functionalities such water‐resistance, poor mechanical and gas barrier, and inadequate thermal properties and flexibility compared to conventional plastic packaging materials. To improve these functional properties, the incorporation of several reinforcement agents is currently being explored such as the use of (nano)fillers (e.g., clay, montmorillonite, lignocellulosic fibers, metal oxides), bioactive compounds, cross‐linkers, or plasticizers creating composite bioplastics (Castro‐Muñoz, Karaça et al., 2023; Castro‐Muñoz, Kharazmi et al., 2023; Garavand et al., 2022; Siddiqui et al., 2024; W. Zhang et al., 2023). When bioactive compounds (antimicrobials, antioxidants, flavors, and colors) are added to bioplastics, these compounds are expected to be released into food to provide certain properties and extend the shelf‐life of food. Therefore, such packaging can also be considered under the overarching category of active packaging (next section) (Diaz‐Montes & Castro‐Muñoz, 2021; Mellinas et al., 2016). Encapsulation of bioactive compounds can enhance their bioavailability and stability (Castro‐Muñoz, Kharazmi et al., 2023). Siddiqui et al. (2023) used mathematical modeling to explore various encapsulation methods for bioactive compounds and understand their possible release mechanisms. They found that encapsulation in nanocapsules enhanced prolonged bioavailability and improved the release rate of bioactive substances, with the release influenced by multiple parameters such as the solubility of the substances, and the release kinetics.

Bio‐based fibers refer to natural fibers produced by plants or animals such as cellulose or silk. Paper and cardboard are made of natural fibers of bleached or unbleached cellulose, from wood pulp (in 95% of cases), but such materials can also be derived from straw, sugar cane bagasse, and bamboo, among others (Stravens, 2023). Paper and cardboard have been used for food packaging for a long time and tend to be used mainly for dry food. Packaging manufacturers use various techniques to increase the versatility of paper and cardboard (Dai et al., 2021; Mujtaba et al., 2022). For instance, a variety of chemical agents can be applied to paper and cardboard to make them suitable for wet food and food with high‐fat content.

As edible materials can be consumed in addition to the food (or beverage) contained within, they potentially offer a combination of sustainability with nutritional properties. Edible food packaging is generally composed of biopolymers or biomacromolecules such as proteins (e.g., gelatin, wheat gluten, and soy protein), polysaccharides (e.g., starch, chitosan, alginate, pectin, carrageenan, and chitosan), and lipids (e.g., beeswax and vegetable oil) extracted from terrestrial or marine plants or animal biomass. Bioactive compounds can be added to this packaging to reinforce functional properties. Incorporation of bioactive compounds, extracted from industrial waste, into edible packaging is an active area of research (Mellinas et al., 2016). Composite edible films formed by the association of both hydrophilic (polysaccharides and proteins) and hydrophobic (lipids) compounds can be used to improve the functional properties of packaging (Echegaray et al., 2023; Kumar et al., 2022; Perera et al., 2021). Edible packaging certainly offers certain benefits, particularly in terms of reducing pollution waste from packaging and replacing fossil‐based plastic packaging. However, their technical scope of application remains limited by their suitability toward very specific food matrices, inherent shelf‐life and storage condition constraints, and inertness toward gaseous exchanges or microbiological contamination. Moreover, this alternative is not yet at a state of industrial maturity to replace the volume of alternative packaging needed to have an impact on reducing dependence on conventional packaging. In fact, based on a survey carried out by the European Commission (2023), edible packaging is not considered a significant alternative for food packaging.

While bio‐based materials are considered to have lower environmental impacts as compared to conventional food packaging, it is important to consider the true environmental impacts of large‐scale production of such materials as well as the short lifecycle of packaging in general (Ghosh & Jones, 2021). Moreover, it is important to point out that some petrochemical‐based polymers are biodegradable while not all bio‐based polymers biodegrade. A material is biodegradable if under certain conditions of temperature, pH, oxygen content, and humidity, the material undergoes chemical processes to form carbon dioxide, water, and biomass due to microbial activity (Emadian et al., 2017; Lucas et al., 2008). Under anaerobic conditions (e.g., in biogas plants), methane is also formed. Therefore, to encourage large‐scale biodegradability and prevent littering, packaging waste will have to be properly collected and managed by being routed to specialized recycling facilities, which may not always be compatible with the existing waste management options. Clear labeling of bioplastics and awareness‐raising campaigns will be needed to ensure proper treatment and disposal of bioplastics to prevent exacerbation of issues linked to littering (EEA, 2020).

#### 3.3.2 Potential food safety hazards

The composition of bio‐based materials can vary greatly based on the source (vegetable or animal‐derived) of the material. This potential variability from one batch to another makes it difficult to characterize all possible hazards associated with bio‐based materials.

The food safety hazard profiles of some bio‐based materials can be similar to the feedstock the materials are derived from, for instance, bioplastics derived from agricultural biomass may have contaminants commonly associated with the latter such as agrochemical residues. Therefore, the array of hazards can be quite different from those usually found in conventional packaging. Some potential hazards linked to bio‐based materials include (Cavazza et al., 2022; FAO, 2021c; Fera Science Ltd, 2019; van der A, 2020):

‐ inorganic contaminants such as heavy metals (e.g., lead, cadmium, and mercury),
‐ persistent organic pollutants (polycyclic aromatic hydrocarbons, dioxins or polychlorinated biphenyls),
‐ contaminants arising from processing, such as thermal processing resulting in the formation of acrylamide in the presence of proteins,
‐ residues from agrochemicals such as pesticides or veterinary medicines,
‐ natural toxins such as phytotoxins (e.g., pyrrolizidine alkaloids), mycotoxins (e.g., aflatoxins), or algal biotoxins
‐ allergens,
‐ phytoestrogens, and
‐ microbial hazards.

While the ability of the above‐mentioned hazards to migrate from the packaging material into food is not fully understood, there are some reported health impacts associated with them including genotoxicity, carcinogenicity, organ toxicity, and effects on the hormonal system.

Allergenicity is an issue that can be associated with bio‐based FCM due to the presence of known and suspected allergens, such as latex proteins, Hev b1, Hev b3, Hev b5, and Hev b6.02 from the rubber tree. However, currently, limited information is available on this issue, including how processing methods used in the production of packaging alter allergenicity or what is the potential for the transfer of these allergens into food (Fera Science Ltd, 2019; Packaging Europe, 2021). Some of the known sources of potential allergenic proteins in bio‐based packaging are derived from (Cavazza et al., 2022):

‐ plants: soybeans, quinoa, sunflower, rubber tree (latex), pea, corn, wheat (gluten), seaweed; or
‐ animals: chitin and chitosan from shellfish, eggs, milk, bovine hide (gelatin), feather.

Most proteins from these sources can be used in films or coating of films in food packaging. The possible transfer of latex proteins from rubber‐based food packaging to food has been reported (BBC News, 2006; Topping et al., 2006) when natural latex is used as film coatings to replace the plastic layer. Different types of allergic reactions can occur upon contact with latex proteins, such as Type I immediate allergy (due to latex proteins), and Type IV delayed allergy contact dermatitis (due to chemical additives within processed rubber products). Cases of urticaria have been reported after direct contact with latex‐based wrapping of chocolate bars (Hughes, 2008; Mak et al., 2005). Among bio‐based materials, the use of chitosan, derived from chitin, is used in the form of flexible films or coatings. This application is especially promising due to its potential antimicrobial activity (Liu et al., 2022). Incomplete deproteinization of chitin during the purification process may lead to the presence of allergenic proteins, such as tropomyosin (the main allergen in seafood) in the final material (Muzzarelli, 2010). Consideration of allergenic potential of bio‐based materials is important, especially when used for edible packaging.

Some bio‐based materials used for food packaging have limited barrier and mechanical properties, necessitating blending with synthetic polymers or production with synthetic additives to strengthen functionalities. Therefore, depending upon the composition of bio‐based materials, certain substances commonly found in conventional petroleum‐based packaging could also be present in bio‐based materials, potentially posing health risks (Zimmermann et al., 2019, 2020). A study by Osorio et al. (2021) showed that polyester oligomers, both linear and circular (such as oligomers of adipic acid, propylene glycol, dipropylene glycol, and isobutanol) can migrate from biopolymers (made from polylactic acid and starch, and often blended with polyester resins to improve functional properties) to food (Osorio et al., 2021). These polyester oligomers are considered NIAS, and there is little information available on the adverse health impacts of these substances.

In terms of edible packaging, food safety is a major concern as such packaging tends to be in direct contact with the external environment and is meant to be directly ingested. Therefore, they need to be produced under the same requirements of Good Manufacturing Practices for food and evaluated for safety at the same level as food ingredients. As noted above, the allergenicity of some materials used for edible packaging is an area that deserves attention. Macroalgae is a popular material of choice for creating edible packaging, and all possible food safety issues linked to macroalgae will need to be considered. A recent report by FAO elucidated the various food safety issues associated with macroalgae, which include various microbiological, chemical (including the potential presence of heavy metals, iodine, persistent organic pollutants, and phycotoxins), the risk of allergenicity, and physical hazards (such as microplastics) (FAO, 2022b).

Bio‐based materials may also include bio‐based nanomaterials such as cellulose nanocrystals, cellulose nanofibers, and chitin nanocrystals (Wang et al., 2022). Food safety risks associated with nanomaterials are discussed in the section on nanotechnologies.

#### 3.3.3 Regulation

In the European Union, bioplastics made of macromolecules that are chemically modified, or those manufactured by microbial fermentation need to be compliant with Annex I of the European Plastic Regulation, which lists the monomers that can be used in the production of bioplastics. These include ethylene, propylene, lactic acid (PLA), and monomers used for polyhydroxyalkanoate, among others (Fera Science Ltd, 2019). In the United States of America, the term “bioplastic” does not have a formal definition. The US FDA regulates FCM as indirect food additives. In case of use of a polymer for which an FDA position has not been established, a Food Contact Notification is required for the FDA to review the safety of the materials.

For paper and cardboard, there are no harmonized regulations in the European Union. However, as with any other FCM, paper and cardboard need to be compliant with regulation 1935/2004. At the national level, the German Federal Institute for Risk Assessment (BfR) published recommendations for paper and cardboard used as FCM (BfR, 2023). In the United States of America, paper and cardboard in contact with food must be compliant with the requirements listed in 21CFR§176.170.

While edible packaging is not currently regulated, the packaging constituents must be considered as food ingredients or additives to safeguard the quality of the food and consumer health. In the United States of America, the constituents of edible packaging need to have a Generally Recommended As Safe status to be compliant with FDA regulations (Ajesh et al., 2022).

### 3.4 Active and intelligent materials

#### 3.4.1 Description

Aided by digital innovation and scientific advances, packaging technologies, over the last decade, have gone beyond the primary roles of packaging to also prolong product shelf‐life during transportation and storage while controlling the overall quality of food contained (Ghoshal, 2018).

Such technologies can be broadly split into two main categories: intelligent packaging and active packaging (EFSA, 2009). While conventional packaging is developed to be as inert as possible to food with adequate functional barriers minimizing the exchange with the food contained, active and intelligent packaging in fact aims at deliberately interacting with food or the environment around the contained food product (Dainelli et al., 2008). Different types of materials can be used as active and intelligent materials: plastics, paper, metals, or a combination of these materials. The substances used in these materials can be derived from different sources such as bacterial (e.g., *Carnobacterium maltaromaticum* used as time‐temperature indicator), synthetic (e.g., polyacrylic acid, sodium salt, cross‐linked used as liquid absorber), mineral (e.g., iron used as oxygen scavenger), or bio‐sourced (e.g., curcumin used as pH indicator) (EFSA, 2013a, 2014; Knutsen et al., 2018; Roy et al., 2022). In addition, the application of nanotechnology is also widely envisaged as part of these advances in packaging advances to enhance their physicochemical properties.

The primary purpose of intelligent packaging is to “detect and inform” by monitoring the status of the food product, measuring various factors within and around the packaging followed by the interpretation and relay of information, via data carriers, indicators, and sensors (e.g., ripening indicators, gas detectors, and radio frequency identification devices) embedded in the packaging. This information is then sent to an external interface, that allows either consumers to make decisions on the safety and quality of the product or in an operational context to inform actors within the supply chain of the possible adaptations to be made upstream where necessary (Han et al., 2018; Vanderroost et al., 2014).

Active packaging, on the other hand, can dynamically respond to the environment within and around food packaging, or to the information relayed by intelligent packaging technology (e.g., sensors). Such packaging is used to actively adapt to changing conditions by either absorbing or releasing various agents that maintain the safety and quality of food. These agents can be oxygen scavengers, moisture scavengers, and ethylene absorbers in nonmigratory active packaging systems and carbon dioxide emitters, antimicrobial agents, antifungal agents, and antioxidants in active releasing packaging systems (Ahmed et al., 2022).

While such technologies offer interesting features for food preservation and reduction of food loss, there are issues from an environmental sustainability standpoint due to the lack of recycling streams built for these materials and single‐use sensors that are embedded in them (Vanderroost et al., 2014).

#### 3.4.2 Potential food safety hazards

Unlike other FCM, in active packaging, certain substances are intended to be released into food in a controlled fashion. Consequently, the safety associated with the released substance(s) must be assessed during packaging development. Substances used as components of active and intelligent materials must not go beyond safe levels of exposure for the consumer, as such the safety profile of released substances will need to be fully defined. The manufacturer must ensure that the available toxicological data are sufficient for the relevant safety assessment agency to conclude on the safety of the substances prior to their use in a marketed product. If different substances within active or intelligent packaging are meant to interact, these interactions should also be considered in the safety assessment of the packaging (EFSA, 2009).

The other potential safety risks for active and intelligent materials are associated with the specificity of the materials used (plastic, metals, glass, recycled materials, bio‐based materials, etc.) and the associated monomers and additives. In addition, active and intelligent packaging often rely on nanotechnologies. Potential food safety issues associated with the application of nanotechnology in active and intelligent packaging are described in the section below.

#### 3.4.3 Regulation

In the European Union, Regulation No 450/2009/EC and accompanying guidance for petitioners from EFSA have allowed the establishment of specific requirements for the use and authorization of active and intelligent materials intended to come into contact with food. These are used in conjunction with EU Regulation 1935/2004 that allows the application of materials with agents that could migrate into foods, opening the pathway for active packaging to be used in the European Union.

In the United States of America, active and intelligent materials are regulated under the FDA's regulatory framework and are subject to the same requirements as all food contact substances. Materials used in food contact applications are subject to premarket regulatory clearance in the country if deemed as “food additives.” However, manufacturers need to account for any additional migrants, decomposition byproducts, or impurities that may occur because of chemical activity in the active packaging material during its storage and shelf life. In case antimicrobials are used, they also must be registered with the US Environmental Protection Agency (Misko, 2022).

### 3.5 Nanotechnologies applied across food packaging solutions

#### 3.5.1 Description

While the application of nanotechnology does not represent a category of food packaging on its own, it can be integrated within most types of materials used in food packaging, including active and intelligent packaging, creating the growing field of nano‐food packaging that offers a variety of potential functionalities. Nanotechnology can be used to enhance barrier and mechanical properties, and maintain food safety and quality, while extending product shelf life, through the release of antimicrobials, antioxidants, flavors, enzymes, and neutraceuticals, among others (Sharma et al., 2017). Moreover, the use of certain nanomaterials derived from renewable sources such as cellulose or chitin nanocrystals can also reduce the dependence on petroleum‐based packaging (Emamhadi et al., 2020). Nanotechnologies applied to food packaging fall into three main categories: nanoparticles (e.g., titanium dioxide, silicon dioxide, silver nanoparticles, and zinc oxide), nanocomposites (nanoclay, nanoencapsulation, and bionanocomposites), and nanosensors (metal‐based nanosensors, nanobiosensors, nanosmart dust, and nanobarcodes) (Onyeaka et al., 2022).

Various nanoparticles of metal and metal oxides (zinc, titanium, copper, gold, and silver) or inorganic nanoparticles have been shown to be effective in active packaging where they can be used as reservoirs for substances meant for controlled release. These substances can be used for their antimicrobial and/or biocidal activities, or for their role in limiting moisture and gas exchange with the environment (Jafarzadeh et al., 2020). Nanoparticles in FCM can either be part of the IAS added to food packaging for their specific nano functionalities or be present unintentionally in the packaging, for instance, in the case of titanium dioxide when used as a colorant or an opacifer (Silano et al., 2019).

Nanocomposites are hybrid materials, often with multiple phases of which at least one phase in the nanometer range (10–200 nm). Films and coatings incorporated with nanocellulose are used in food packaging for preservation, given their antioxidant and antimicrobial activities. They also provide good barrier properties due to their compactly packed structure (Ahankari et al., 2021; Ashfaq et al., 2022; Perumal et al., 2022). Gelatine nanoparticles, cellulose nanocrystals, and coatings made of chitosan and nano‐silicon dioxide are examples of edible and biodegradable coatings (Ahari et al., 2021).

Nanosensors, usually found in intelligent packaging, have an important role in the storage and transportation of food by being able to sense and signal information about quality, freshness, and chemical, physical, and microbiological changes (Duran & Marcato, 2013). Nanosensors can also alert consumers and other food business actors to food contamination or food spoilage by detecting signatures of chemical contaminants and microbial growth in food products and converting them into observer readable signals, enhancing food traceability. Metal‐based nanosensors (platinum, gold, and palladium) can have multiple applications, such as detecting gas production and color changes in food from spoilage due to changes in humidity, temperature, light, and production of certain toxins (Onyeaka et al., 2022).

#### 3.5.2 Potential food safety hazards

Several food safety competent authorities have determined the possible adverse health effects associated with nanoparticles ingested through food and have provided guidance for risk assessment of nanomaterials in food and the use of nanomaterials in food packaging (EFSA, 2021b; FSANZ, 2016; US FDA, 2018b). However, the migration of nanoparticles from FCM into food and the potential to cause human health consequences once consumed are still not well understood (Froggett et al., 2014; McClements & Xiao, 2017; Störmer et al., 2017). This complicates risk assessments of nanomaterial safety (Bandyopadhyay & Sinha Ray, 2018; Onyeaka et al., 2022). Depending on the composition, size and morphology, solubility, rate of migration, behavior in food, and the amount consumed, certain nanoparticles may be a health hazard in case they are absorbed in the gastrointestinal tract with subsequent distribution and accumulation in various organs (Fröhlich & Fröhlich, 2016). According to literature, some nanoparticles may have adverse effects on the immune system, cause genotoxicity and carcinogenicity, as well as reproductive and developmental toxicity (Shukla et al., 2021; Umezawa et al., 2017). For example, some studies highlight that silver nanoparticles can have possible adverse effects on the reproductive system and on the development of the fetus through the translocation of the nanoparticles from the mother to the fetus (Zhang et al., 2021). Other studies have found that exposure to titanium dioxide nanoparticles may cause inflammation, cytotoxicity, genotoxicity, and cell apoptosis, but there are inconsistencies between different studies (Park et al., 2008; Wani & Shadab, 2020). Adverse effects caused by nanomaterials are expected to be similar compared to their bulk (non‐nano) materials if it can be demonstrated that a nanomaterial loses its particulate nature due to solubilization or as a result of a physical, chemical, or biological process. If this is not the case, the presence of nanoparticles may exert additional toxicological effects due to their specific physicochemical properties, such as larger surface areas and greater surface energies, making them more reactive (EFSA, 2021b). It must be pointed out that most toxic effects after exposure to nanoparticles are documented after inhalation with less toxicological data available after oral exposure (Brohi et al., 2017; Zhang et al., 2021). Therefore, while rapid progress has been made in the application of nanotechnology in food products, the potential for toxicity of nanoparticles and nanomaterials needs further research.

In addition, there is growing concern about the issue of environmental contamination by nanomaterials during the degradation of nanomaterial‐based packaging. The distribution and fate of nanomaterials in the environment and the impact on human health from exposure through the food chain is still unclear (Souza & Fernando, 2016).

#### 3.5.3 Regulation

In the European Union, like other FCM, those made with nanotechnologies must be compliant with Regulation 1935/2004 which sets out the general principles of safety and inertness. For plastics, only titanium nitride is currently authorized for use as nanoparticles as per the European Regulation No10/2011 for the production of plastics and specifies that the nanoparticles must not migrate (EFSA, 2012; European Commission, 2011). In the case of the use of active or intelligent materials, risk assessments are carried out by EFSA on the potential presence of nanoparticles based on all supplied data.

In the United States of America, nanotechnologies used in FCM are regulated by the FDA and are assessed as (indirect) food additives. Manufacturers must obtain premarket approval for indirect food additives unless the substance is already listed as Generally Recognized As Safe (US FDA, 2018a).

### 3.6 Hybrid packaging

#### 3.6.1 Description

Hybrid packaging is developed to be flexible and lightweight by generally using only a thin layer of plastic which tends to be in direct contact with food and a rigid layer of paper/cardboard which allows reinforcement of the overall structure. After the food is consumed, the different layers constituting the packaging can be separated by consumers, operators, or automated machines in recovering facilities, with the cardboard and plastics entering their respective recycling streams. Cardboard can even be composted or reused instead. Another advantage of hybrid packaging is that it provides avenues to expand the application of materials such as paper and cardboard. While it tends to be largely used to contain dry food (e.g., flour, table sugar, pasta), it is now being used in packaging for wet foods (e.g., yogurt cups) without the need to use grease‐proofing and water‐repellent chemicals, some of which are recognized as problematic pervasive substances (e.g., PFAS).

‘Hybrid packaging allows customization of the various FCMs used in the packaging solution. By doing so, it allows sustainability aspects of the different FCMs used to also play a part in these considerations’ with ‘Hybrid packaging allows customization of various FCMs. This allows the opportunity to use sustainability aspects of these FCMs as a criteria for choosing certain FCMs as components of hybrid packaging’.

#### 3.6.2 Potential food safety hazards

In hybrid packaging, the first material in contact with food tends to be thinner than in the case of conventional packaging. Generally, the thicker the material the higher the potential for migration. Therefore, using a thinner layer of materials may be considered favorable for decreasing the possibility of migration. On the other hand, the thinner the layer of material in contact with food, the more likely it is for substances from the outer layers of the packaging (outer cardboard, label, inks used, or adhesives) that are in indirect contact to migrate into food unless a functional barrier is used to prevent this migration from the outer layers (Gerassimidou et al., 2022).

#### 3.6.3 Regulation

There is no specific regulation for hybrid packaging. Each material used in hybrid packaging needs to be compliant with the appropriate regulation governing its intended use in packaging as applicable in the jurisdiction.

## 4 CHALLENGES ASSOCIATED WITH CURRENT ANALYTICAL METHODS AND MIGRATION TESTING USED FOR RISK ASSESSMENT OF FCM

The exposure assessment, essential for the risk characterization process to ensure consumer safety, relies almost exclusively on declarations by food packaging suppliers and the analytical methods used to determine the substances that migrate into food. The IAS in packaging can either be banned or authorized, and if authorized, this may come with or without certain restrictions (e.g., benzophenone is authorized for the manufacturing of plastics with a specific migration limit set at 0.6 mg/kg as per regulations in Europe). In some cases, IAS may not be specifically regulated. Regarding NIAS, as their presence cannot always be predicted, they tend not to be regulated, but in certain jurisdictions, like China and the European Union, there are specific requirements for the assessment and evaluation of NIAS in FCM (European Regulation 10/2011 and China Standard GB 4806.1).

In the European Union, currently, migration testing requirements include food simulants, time, and temperature conditions for overall and specific migrations, and are well defined for plastics (European Commission, 2011). When a substance with a certain restriction on usage or a known/predictable NIAS is declared by the supplier of an FCM, specific migration tests are performed. As food is a complex matrix, the use of appropriate food simulants that mimic food properties but in a simpler environment facilitates the detection of migrating substances. This process occurs under specific conditions of time and temperature to achieve accelerated aging conditions corresponding to the shelf life of the desired food product. For the FCM to be approved, the level of migration obtained needs to be below the specific migration limit, as provided by the regulation for the packaging, to be compliant. Usually, the same test conditions to address migration are also used to screen for NIAS. For FCM meant for repeated use, regulations in the European Union recommend repeating the migration testing three times to ensure that the migration level does not increase. Some limitations in this process include not considering the washing step which can degrade FCM or introduce contaminants from cleaning products. For other FCM (paper and cardboard, coatings, adhesives, silicones, elastomers/rubbers, wood, cork, textiles, glass, etc.) often referred to as non‐harmonized FCM, there are no harmonized rules to follow for migration testing conditions. For paper and cardboard, the recommendation from BfR on “Paper and board for food contact” provides guidance on some food simulants to use depending on the types of substances that need to be analyzed (e.g., cold‐water extraction for aluminum, lead, and cadmium) (BfR, 2023). However, there are no requirements to follow in order to identify and quantify migrating unknown/unpredictable NIAS from paper and cardboard (Nerín et al., 2022). The food contact guidelines for the compliance of paper and cardboard materials and articles published by Confederation of European Paper Industries provide some guidelines to follow by industry type, but they are not always recognized by authorities. In the United States of America, no migration testing is required on the final article, and safety and compliance are based on the properties of the material used. For example, paper and cardboard in contact with food must be compliant with 21CFR176, which lists the substances that can be used and their restrictions. The differential risk management approaches along with the lack in harmonized rules for migration testing is a major limitation in the assurance of safety and consumer protection.

Once migration testing is complete, complex analyses are required to identify and quantify all the potential migrating substances. The analysis of NIAS is particularly challenging as it is difficult to predict all NIAS, and currently, there are no standardized analytical methodologies for nontargeted screenings. Nowadays, NIAS analyses are mostly based on the use of gas chromatography (GC—for volatile and semi‐volatile substances) and high‐performance liquid chromatography (for nonvolatile substances) coupled with mass spectrometry (MS) for identification and quantification. However, the analytical methods used also depend on the food simulant and the type of migrating substances. Besides, not all food simulants are compatible with all types of packaging materials (Nerín et al., 2022).

Identification of substances also depends on libraries of molecules available to cross‐check which substances are present among the density of peaks obtained from chromatography with identification sometimes limited to the substance class, such as PET oligomers (Schreier et al., 2023). Available libraries are not always exhaustive and do not always enable the identification of all molecules present in the sample. In addition, there are limitations due to the unavailability of pure or certified standards for the confirmation of the full identity and quantification of compounds.

As science progresses, analytical methods are improving, thereby providing higher sensitivities by attaining lower limits of detection and quantification, resulting in an increased detection of potentially migrating substances including NIAS. However, despite the wide spectrum of analytical techniques, there are still significant limitations. Nonharmonized protocols involving different sample preparation techniques can lead to different analytical results, raising potential concerns that may or may not be justified. Prior knowledge on potential migrating substances, especially NIAS, is crucial for the selection of appropriate analytical methods (Nerín et al., 2022). Limits of detection needed for regulatory, or safety requirements are not always achievable, or substances are not always fully characterized in a NIAS screening, especially with generic screening approaches. As an example, based on the newly set tolerable daily intake for BPA, as established by EFSA (EFSA, 2023), the theoretical acceptable migration levels would fall in the parts per trillion range, while it used to be in the part per million or hundreds of parts per billion range. However, this will require a huge step forward in analytical sensitivity, and the current available methods do not reach levels of detection that low. For certain substances, such as primary aromatic amines, formaldehyde, acetaldehyde, PFAS, or organotin compounds, specific methods are needed to detect (within required sensitivity) them. Traditionally, the management of FCM focuses on the analysis of substances considered individually. Such methodologies are not always fit for purpose given the emergence of contaminant families, such as PFAS, which is a large family of over 10,000 substances. The detection and quantification of PFAS can be highly variable depending on the food or food simulant matrix used in the analysis. While several analytical methods have been developed for the analysis of some specific PFAS members, they are only a small fraction of the very large family of substances. The other option is to use a nonspecific method to measure total fluorine, but this can lead to an overestimation as fluorine may be present in other fluorinated compounds or as inorganic fluorine (Genualdi et al., 2022; Jovanović et al., 2024; Ramírez Carnero et al., 2021; Rawn et al., 2022). Another issue related to PFAS is that contemporary policies can sometimes by driven by hazards identified rather than assessed risks, possibly imposing precedents of regulatory requirements, and in some cases even before the establishment of appropriate analytical capabilities.

With respect to the new packaging alternatives described in the previous section, it is possible to expect new types of molecules to migrate from the packaging, depending on the materials used. Analytical methods will need to be improved/expanded to detect these “new” types of molecules, such as proteins of higher molecular weight and capable of causing allergic reactions. As current analytical methods (typically GC‐MS or LC‐MS) do not detect molecules above 800/1000 Da, proteins of higher molecular weight may remain undetected. Molecules with a molecular weight above 1000 Da typically do not present a safety concern as they are not absorbed in the gastrointestinal tract (EFSA, 2016b). However, in the case of allergenicity, local contact of proteins with the mucosal membrane may be sufficient to cause an immune response. A potential solution would be to identify and quantify these proteins by coupling migration testing with other methods such as enzyme‐linked immunosorbent assay or polymerase chain reaction. Whether natural compounds generally present in bio‐based materials (such as natural toxins) can be detected with current analytical methods is a topic of further research.

In case of unknown or “not fully identified” substances, for which genotoxicity cannot be ruled out, the acceptable migration level is considered to be 0.15 µg/kg based on the threshold of toxicological concern (TTC) for genotoxic substances (EFSA, 2019). In many cases, this low migration level can be a challenge for the analytical capabilities, particularly with nontargeted approaches. Ruling out genotoxicity would allow for a higher TTC value and a higher corresponding acceptable migration limit. In these cases, bioassays may be used to exclude the genotoxicity concern. Indeed, after migration analyses, the mixture of migrating substances can be directly tested in genotoxicity bioassays to determine if the mixture contains potential genotoxic substances. Schilter et al. (2019) reviewed the values and limitations of in vitro bioassays to support the application of the TTC approach to prioritize unidentified chemicals in FCM. They concluded that the miniaturized Ames test (i.e., Ames test adapted to mixtures and smaller concentrations) appeared to be the most suitable genotoxic bioassay for migrating substances from FCM, since it specifically detects DNA‐reactive mutagens. However, validation studies may be needed to assess if these miniaturized assays can be used as an alternative to the standard Ames test, as the miniaturized versions are currently not widely accepted by regulatory authorities (OECD, 2022a). Another promising genotoxic bioassay adapted for migrating substances from FCM is the high‐performance thin‐layer chromatography coupled with the planar SOS Umu‐C bioassay, which can detect low levels of mutagens and genotoxins in complex mixtures. The results from this bioassay could be followed by a classic Ames assay to confirm the mutagenicity of the substance(s) (Debon et al., 2022; Meyer et al., 2021, 2023).

## 5 CONCLUSION AND FUTURE PERSPECTIVES

In this review, we discussed the emerging trends that are shaping the food packing landscape, some recent alternatives as well as the main food safety concerns associated with them. The predominant trends reported are the growing awareness of the environmental impacts of conventional packaging waste, consumer perceptions about the health impacts of food packaging, and an expected increase in the use of food packaging in the coming years. While ensuring consumer safety remains the main priority for the food packaging industry, today the environmental impacts of postuse food packaging cannot be ignored. The packaging alternatives presented include new materials (recycled, bio‐based), new ways of using (reusable packaging) or assembling existing materials (hybrid packaging), and new technologies to improve the properties of existing materials (nanotechnologies, active and intelligent packaging).

It is essential to identify the potential hazards associated with these (new) packaging alternatives to anticipate any risks to consumer safety. While some potential hazards are common to conventional packaging, others can be specific to the new alternatives. The potential migration of additives, nanomaterials, or degradation products can be common between conventional and new packaging materials due to the presence of synthetic additives used to reinforce packaging properties. The potential hazards specific to new alternatives include the presence of proteins and risk of allergenicity in bio‐based materials, microbiological and chemical contaminants in reusable packaging, and the presence of certain substances in recycled materials that are not meant for FCM, among others. Advances in research and analytical methods allow us to better characterize the potential migrating substances from packaging and their associated hazards, improving our knowledge of the potential safety risks associated with food packaging. As the application of new FCM steadily grows, analytical capabilities will need to be constantly improved to cover the diversity of substances used (e.g., proteins). Overall, while materials and conditions of use of food packaging have drastically evolved in the last 30 years, the main food safety risk for consumers remains the ability of substances to migrate from the packaging into food resulting in consumer exposure. These substances must be adequately assessed to ensure that they do not pose a health risk for consumers. This task, however, is complicated by the lack of harmonized regulatory requirements globally, compounding challenges for all stakeholders in the food chain, including the food industry.

The current global regulatory landscape for FCM is very diverse depending on the type of materials and the geographical areas, with rules aimed at ensuring safety drastically varying across countries and regions. This can lead to differences in the interpretation of consumer safety assessments and hinder international trade. Differences in regulatory frameworks can also curb businesses from developing novel technologies and new types of packaging, thus potentially curbing innovations that ensure longer shelf life with far reduced impact on the environment. International guidance, from authoritative bodies like the Codex Alimentarius Commission, on the principles for safe food packaging can help drive global harmonization and play a crucial role in ensuring a consistent and science‐based framework for the safety and compliance of FCM while also facilitating the development of sustainable packaging solutions (FAO & WHO, 2023).

Finally, there are knowledge gaps in assessing the environmental impacts of packaging alternatives—how they are produced, distributed, used, and disposed of and therefore will need further research. A One‐Health approach that combines human, animal, and environmental health outcomes could be an appropriate way to assess new food packaging. It would involve assessing the risks and determining the impacts on humans, animals, and the environment together, thereby supporting the development of future packaging in the most positive and sustainable way possible.

## AUTHOR CONTRIBUTIONS

**Charlene Lacourt**: Conceptualization; investigation; writing—original draft. **Keya Mukherjee**: Investigation; writing—review and editing; conceptualization. **Jossie Garthoff**: Conceptualization; writing—review and editing. **Aaron O'Sullivan**: Conceptualization; supervision. **Leo Meunier**: Conceptualization; supervision. **Vittorio Fattori**: Conceptualization; supervision.

## CONFLICT OF INTEREST STATEMENT

The authors declare no conflicts of interest.

## DISCLAIMER

The views expressed in this publication are those of the authors and do not necessarily reflect the views or policies of the Food and Agriculture Organization of the United Nations.
